# Association between body mass index and ready-to-eat food consumption among sedentary staff in Nay Pyi Taw union territory, Myanmar

**DOI:** 10.1186/s12889-020-8308-6

**Published:** 2020-02-10

**Authors:** Thin Zar Thike, Yu Mon Saw, Htin Lin, Khin Chit, Aung Ba Tun, Hein Htet, Su Myat Cho, Aye Thazin Khine, Thu Nandar Saw, Tetsuyoshi Kariya, Eiko Yamamoto, Nobuyuki Hamajima

**Affiliations:** 1 Department of Food and Drug Administration, Ministry of Health and Sports, Nay Pyi Taw, Myanmar; 2 Department of Healthcare Administration, Nagoya University, Graduate School of Medicine, 65 Tsurumai-cho, Showa-ku, Nagoya, 466-8550 Japan; 3 Nagoya University Asian Satellite Campuses Institute, Nagoya, Japan; 4 Directorate of Medical Service, Nay Pyi Taw, Myanmar; 5 Department of Preventative and Social Medicine, University of Medicine, Mandalay, Myanmar; 6 Department of Public Health, Ministry of Health and Sports, Nay Pyi Taw, Myanmar; 7 Department of Community and Global Health, Graduate School of Medicine, the University of Tokyo, Tokyo, Japan

**Keywords:** Ready-to-eat food, Sedentary staffs, Physical exercise, Nay Pyi Taw, Myanmar

## Abstract

**Background:**

Ready-to-eat (RTE) food consumption has become popular in the working community with the increase in full-time jobs and the limited time to prepare food. Although RTE food is essential for this community, its consumption causes obesity. In Myanmar, obesity is a modifiable risk factor for non-communicable diseases, causing increases in morbidity and mortality. This study aimed to identify the association between body mass index (BMI) and RTE food consumption among sedentary staff in Nay Pyi Taw Union Territory, Myanmar.

**Methods:**

A cross-sectional study was conducted in 2018, in which 400 respondents participated in face-to-face interviews. The study area was selected using simple random sampling and drawing method. Measuring tape and digital weighing scale were used to measure the height and weight of the respondents. BMI was calculated by dividing the weight by height squared (kg/m^2^). Overweight and obesity were categorized by World Health Organization cut-off points. The collected data were analyzed using multiple logistic regression to estimate the adjusted odds ratio (AOR), and the 95% confidence interval (CI).

**Results:**

This study revealed that sedentary staff who consumed RTE food once or more per month were nearly five times more likely to be overweight and obese (AOR = 4.78, 95% CI 1.44–15.85) than those who consumed RTE food less frequently. In addition, five factors namely being older than 32 years (AOR = 3.97, 95% CI 1.82–8.69), preference for RTE food (AOR = 8.93, 95% CI 2.54–31.37), light-intensity of physical exercise (AOR = 3.55, 95% CI 1.63–7.73), sedentary leisure activities (AOR = 3.32, 95% CI 1.22–9.03), and smoking (AOR = 5.62, 95% CI 1.06–29.90) were positively associated with overweight and obesity.

**Conclusion:**

Frequent consumers of RTE food and less physically active sedentary staff were more likely to be overweight and obese. This study highlights the urgent need to raise awareness regarding healthy lifestyle behaviors among the working community to reduce the burden of obesity-related chronic diseases. Moreover, sedentary workers should be aware of the food-based dietary guidelines of the country. Policy makers should strictly enforce nutritional labeling of RTE food, and strictly prohibit over-branding of RTE food.

## Background

The trend of ready-to-eat (RTE) food consumption has been increasing significantly globally due to rapid urbanization, busy lifestyles, and convenient access to fast food facilities [1, 2]. Due to their fast-paced lives, people prefer unhealthy RTE food to healthy home-made food, as it is affordable, readily accessible, and easy to prepare for everyone, especially for people in dual-income families [3–6]. Frequent and excessive RTE food consumption leads to obesity since it is high in calories, fats, and salts [7].

RTE food is defined as “a plant- or an animal-derived food that has to be frozen, cooked, and processed before it can be directly consumed and requires a very minimal time of preparation involving boiling or reheating before consumption” [8]. In the United States, 36% of adults are reported to consume RTE food daily [9]. RTE food consumption has been increasing in Asia [10]. RTE food has an important part in high-income countries, as well as middle- and low-income countries, including Southeast Asia [11]. Unhealthy foods such as RTE food and a physically inactive lifestyle are not only the major risk factors for chronic non-communicable diseases (NCDs), but are also the main causes of obesity [12].

Obesity has an increasing trend and its prevalence has doubled worldwide since 1980 [13]. According to a global estimation study, more than 650 million people were estimated to have adult obesity and 1.9 billion people were estimated to be overweight in 2016 [14]. Overweight and obesity have become global public health problems and are some of the top leading causes of death [15]. World Health Organization (WHO) estimated that 4.5 million deaths worldwide were attributable to the consequences of overweight and obesity in 2016 [16]. Obese people are prone to have chronic diseases such as cardiovascular diseases, hypertension, and diabetes mellitus, leading to increased morbidity and mortality. Obesity-related chronic diseases are a burden for the country [17]. For example, 5 to 10% of the country expenditure is spent on obesity-related chronic diseases in the United States [18]. Diabetes mellitus, along with hypertension and physical inactivity, is still a major risk factor for cardiovascular diseases. The cost of treating these risk factors are far less than treating their consequences [19–22].

The rates of overweight and obesity have increased in developing countries [23], where the prevalence has increased from 857 million in 1980 to 2.1 trillion in 2013, which was equivalent to 60% of the global population [24]. In the Asia-Pacific region, the prevalence of overweight was 50% in 2017 [25]. In Myanmar, the estimated prevalence of overweight increased from 24% in 2008 to 28% in 2015, whereas the prevalence of obesity increased from 6% in 2008 to 13% in 2015, implying an alarming rate of increase [26, 27]. In 2012, 59% of total deaths occurred due to NCDs in Myanmar [28].

In Myanmar, RTE food consumption affects the country’s economy. With the growing economic development, foreign investment in Myanmar has been expanding, especially in the food and beverages sector, an important component of the country’s economy. Increasing demand for RTE food contributes to an increasing amount of imported food and beverages accounting for nearly 50% of the total import of the country. Local production of RTE food has been increasing as well. More than 60% of the family expenditure was being used for food [29, 30]. Along with economic, political, and social context changes, disease patterns have also been shifting from communicable to NCDs [31, 32]. Consumption of unhealthy food leads to obesity, one of the major risk factors for NCDs in Myanmar. Policy makers have targeted to promote healthy workplaces by reducing modifiable risk factors such as unhealthy diet, smoking, alcohol drinking, and sedentary activities [33, 34].

Most of the full-time staff working at a public institution in urban cities are sedentary, and their lifestyles cause them to consume RTE food. However, studies focusing on unhealthy diet consumption and physical activity status among sedentary workers are limited. This study aimed to identify the association between BMI and RTE food consumption among sedentary staff in Nay Pyi Taw Union Territory, Myanmar. This study is expected to support policymakers in implementing effective health promotion programs for reducing the prevalence of overweight and obesity among the working community, and for initiating healthy lifestyle behaviors at workplaces which, in turn, will reduce the burden of NCDs in the country.

## Methods

### Study subjects

A cross-sectional study was carried out at a public institution in Nay Pyi Taw Union Territory, Myanmar, from July to September 2018. Among the 25 public institutions in Nay Pyi Taw, one institution was selected by a drawing method first. Second, four departments from the institution were randomly selected by the same method. Third, 100 respondents aged between 18 and 60 years who attended the office on the study day were randomly recruited from the attendance list of the office to get the required sample. Pregnant women and lactating mothers were excluded from the survey. In total, 400 respondents participated in this study.

### Data collection

Data were collected using pretested semi-structured questionnaires in Myanmar language. The research team included a leading researcher and three trained research assistants. The questionnaires included two main sections: 1) background characteristics and 2) RTE food consumption (Additional file 1). The height and weight of the respondents were measured by the trained research assistants, using a Seca measuring tape and a digital weighing scale, respectively. The weighing scale was calibrated before usage. Bodyweight was recorded to one decimal point. Standing body height was recorded to one decimal point with the respondents in barefoot. The respondents were informed about the study objectives and the content of the study prior to the interview. The respondents were interviewed face-to-face by the research team members. Each interview took about 30 to 40 min to complete, including the weight and height assessments.

### Statistical analysis

The collected data were recoded and analyzed by using the Statistical Package for Social Science (SPSS) software version 21.0 (IBM SPSS Inc). To present the characteristic of the respondents, frequency distribution and percentage were used. BMI was calculated by dividing weight in kilogram by height in meter squared. It was categorized by using WHO cut-off points: BMI 25–29.9 kg/m^2^ as overweight, and BMI ≥ 30 kg/m^2^ as obesity [35]. Chi-square test was used to compare categorical data. A multiple logistic regression analysis was used to estimate the adjusted odds ratio (AOR), and 95% confidence interval (CI). In this study, a *p*-value of less than 0.05 was considered as statistically significant.

## Results

Table 1 presents the background characteristics of sedentary staff according to sex. Among the 400 respondents, 84.0% were female, and the rest were male. The mean age was 32 years and most of the respondents (54.8%) were aged between 18 and 30 years at the time of the survey. Respondents working overtime for more than 5 h per month were 26.3%. A majority of the respondents (80.0%) were clerical and administrative staff, ranked below the gazetted officer level. Nearly one-third of the respondents were married, but 70.2% had no children. Among the respondents, 19.8% had a medical history of NCDs such as hypertension, diabetes mellitus, and ischemic heart diseases.

**Table 1:** Background characteristics of sedentary staffs (*N* = 400)

Characteristics	Male (*n* = 64)		Female (*n* = 336)		Total (*N* = 400)	
	n	%	n	%	N	%
Age (years)						
18–30	31	48.4	188	56.0	219	54.8
31–40	14	21.9	108	32.1	122	30.5
41–50	13	20.3	28	8.3	41	10.2
51–60	6	9.4	12	3.6	18	4.5
Designation						
Other rank	41	64.1	279	83.0	320	80.0
Officer and above	23	35.9	57	17.0	80	20.0
Average monthly overtime hour						
Less than or equal to five	40	62.5	255	75.9	295	73.7
More than five	24	37.5	81	24.1	105	26.3
Marital status						
Others^b^	35	54.7	228	67.9	263	65.7
Married	29	45.3	108	32.1	137	34.3
Presence of children						
No	38	59.4	243	72.3	281	70.2
Yes	26	40.6	93	27.7	119	29.8
Number of children						
Single	47	73.4	301	89.6	348	87.0
More than one	17	26.6	35	10.4	52	13.0
Type of family						
Nuclear	56	87.5	303	90.2	359	89.7
Extended	8	12.5	33	9.8	41	10.3
Monthly family income (MMK^c^)						
Less than or equal to 450,000	38	59.4	239	71.1	277	69.2
More than 450,000	26	40.6	97	28.9	123	30.8
Presence of housemaid						
Present	4	6.3	7	2.1	11	2.8
Absent	60	93.7	329	97.9	389	97.2
Role of cooking in family						
Main	9	14.1	185	55.1	194	48.5
Supportive	55	85.9	151	44.9	206	51.5
Medical history of NCDs^a^						
No	47	73.4	274	81.5	321	80.2
Yes	17	26.6	62	18.5	79	19.8

^a^NCDs: non-communicable diseases (hypertension, diabetes mellitus and ischemic heart diseases)

^b^Others: single, divorced, separated, widow/widower, ^c^MMK-Myanmar kyats (1USD = 1512MMK on 22.4.2019)

Table 2 shows the lifestyle characteristics of sedentary staff. Of 400 respondents, 3.5% smoked, 6.8% consumed alcohol, and 49.5% had no habit of regular physical exercise. About half of the respondents (50.5%) did regular physical exercise**,** but most of them (91.3%) exercised less than 150 min per week. A majority (75.8%) of the respondents performed sedentary leisure activities such as watching TV, surfing the internet, and reading, while 12.7% used active transport such as going on foot or by bicycle. Regarding BMI, 14.2% of respondents belonged to the overweight and obese category.

**Table 2:** Lifestyle characteristics of sedentary staffs (*N* = 400)

Characteristics	Male (*n* = 64)		Female (*n* = 336)		Total (*N* = 400)	
	n	%	n	%	N	%
Smoking						
No	52	81.2	334	99.4	386	96.5
Yes	12	18.8	2	0.6	14	3.5
Alcohol drinking						
No	44	68.7	329	97.9	373	93.2
Yes	20	31.3	7	2.1	27	6.8
Habit of regular exercise						
No	27	42.2	171	50.9	198	49.5
Yes	37	57.8	165	49.1	202	50.5
Average weekly physical exercise (in minutes)						
More than or equal to150	10	15.6	25	7.4	35	8.7
Less than150	54	84.4	311	92.6	365	91.3
Type of physical exercise						
No and light intensity	39	60.9	205	61.0	244	61.0
Moderate and high intensity	25	39.1	131	39.0	156	39.0
Leisure activity						
Non-sedentary	12	18.7	85	25.3	97	24.2
Sedentary	52	81.3	251	74.7	303	75.8
Travel with job						
No	19	29.7	133	39.6	152	38.0
Yes	45	70.3	203	60.4	248	62.0
Distance between home and other places (in miles)						
More than one	20	31.3	130	38.7	150	37.5
Less than or equal to one	44	68.7	206	61.3	250	62.5
Transportation facility						
Others^b^	57	89.1	292	86.9	349	87.3
On foot/bicycle	7	10.9	44	13.1	51	12.7
^**a**^BMI (kg/m^2^)						
< 25	54	84.4	289	86.0	343	85.8
25–29.9	9	14.1	37	11.0	46	11.5
≥ 30	1	1.6	10	3.0	11	2.7

^a^*BMI* body mass index;

^b^Others: motorbike, car, and public transport

Table 3 shows RTE food consumption of sedentary staff. All respondents consumed RTE food, but the frequency of consumption was quite different. A majority of respondents (73.3%) consumed RTE food more frequently. The most consumed types of RTE food were confections (91.3%), instant-mixes (75.0%), non-alcoholic beverages (59.8%), and instant noodles (30.3%). The vast majority of respondents (84.0%) consumed RTE food for more than 1 year. Two-thirds of them (66.5%) ate RTE food on their free time. Among them, 80.3% had a habit of eating out for RTE food.

**Table 3:** Ready-to-eat food consumption of sedentary staffs (*N* = 400)

Characteristics	Male (*n* = 64)		Female (*n* = 336)		Total (*N* = 400)	
	n	%	n	%	N	%
Frequency of RTE* food consumption						
Less frequently consumed^*^	20	31.2	87	25.9	107	26.7
More frequently consumed^#^	44	68.8	249	74.1	293	73.3
Instant noodle consumption						
Less frequently consumed^*^	44	68.8	235	69.9	279	69.7
More frequently consumed^#^	20	31.2	101	30.1	121	30.3
Sweet confectionaries consumption						
Less frequently consumed^*^	9	14.1	26	7.7	35	8.7
More frequently consumed^#^	55	85.9	310	92.3	365	91.3
Non-alcoholic beverages consumption						
Less frequently consumed*	22	34.4	139	41.4	161	40.2
More frequently consumed^#^	42	65.6	197	58.6	239	59.8
Instant -mix consumption						
Less frequently consumed^*^	11	17.2	89	26.5	100	25.0
More frequently consumed^#^	53	82.8	247	73.5	300	75.0
Habit of eating out						
Not eat out	9	14.1	70	20.8	79	19.7
Eat out	55	85.9	266	79.2	321	80.3
Reason of eating out						
Fun/pleasure	28	43.8	178	53.0	206	51.5
Change/time consuming	36	56.2	158	47.0	194	48.5
Preference for RTE* food						
No	20	31.2	120	35.7	140	35.0
Yes	44	68.8	216	64.3	260	65.0
Duration of consumption						
Less than one year	15	23.4	49	14.6	64	16.0
More than or equal to one year	49	76.6	287	85.4	336	84.0
Timing of consumption						
Dinner	9	14.1	41	12.2	50	12.5
Breakfast/lunch/supper	55	85.9	295	87.8	350	87.5
Free time consumption						
No	25	39.1	109	32.4	134	33.5
Yes	39	60.9	227	67.6	266	66.5
Habit of storage						
No	23	35.9	95	28.3	118	29.5
Yes	41	64.1	241	71.7	282	70.5

^*^ RTE: Ready-to-eat; ^#^More frequently consumed (two to three times per day, once daily, two to four times per week, once per week and one to three times per month); ^*^Less frequently consumed (less than once per month)

AOR and 95% CI of BMI ≥ 25 kg/m^2^ among sedentary staff are shown in Table 4. The respondents older than the mean age 32 years (AOR = 3.97, 95% CI 1.82–8.69) were four times more likely to be overweight and obese than younger ones. The sedentary staff consuming RTE food more than once in a month (AOR = 4.78, 95% CI 1.44–15.58) were nearly five times more likely to be overweight and obese. Moreover, a positive association was found between overweight and obesity, and the preference for RTE food during their free time (AOR = 8.93, 95% CI 2.54–31.37). Similarly, smokers (AOR = 5.62, 95% CI 1.06–29.90) were nearly six times more likely to be overweight and obese than non-smokers. Overweight and obesity (AOR = 3.55, 95% CI 1.63–7.73) were positively associated with light-intensity physical exercise, and sedentary leisure activity in leisure-time (AOR = 3.32, 95% CI 1.22–9.03).

**Table 4:** Odds ratio (OR) and 95% confidence interval (CI) for being overweight and obese BMI ≥ 25 kg/m^2^ among sedentary staffs (*N* = 400)

Characteristics	Unadjusted		Adjusted ^a)^	
	OR 95%CI		OR 95% CI	
Age (in years)				
32 or younger	1	Reference	1	Reference
Above 32	3.51	(1.96–6.27)^***^	3.97	(1.82–8.69) ^**^
Sex				
Male	1	Reference	1	Reference
Female	0.89	(0.42–1.84)	1.99	(0.68–5.79)
Marital status				
Others	1	Reference	1	Reference
Married	1.91	(1.08–3.36) ^*^	1.57	(0.76–3.28)
Designation				
Other rank	1	Reference	1	Reference
Officer and above	2.81	(1.53–5.15) ^**^	1.51	(0.63–3.60)
Smoking				
No	1	Reference	1	Reference
Yes	3.57	(1.15–11.06) ^*^	5.62	(1.06–29.90) ^*^
Frequency of RTE^c^ food consumption				
Less frequently consumed^e^	1	Reference	1	Reference
More frequently consumed^d^	5.69	(2.01–16.12) ^**^	4.78	(1.44–15.85) ^**^
Preference of RTE^c^ food				
No	1	Reference	1	Reference
Yes	11.97	(3.67–39.06) ^***^	8.93	(2.54–31.37) ^**^
Timing of consumption				
Dinner	1	Reference	1	Reference
Others^b^	4.47	(1.06–18.95) ^*^	0.26	(0.05–1.31)
Intensity of physical exercise				
Moderate/vigorous	1	Reference	1	Reference
Light	2.17	(1.14–4.12) ^**^	3.55	(1.63–7.73) ^**^
Leisure activity				
Non-sedentary	1	Reference	1	Reference
Sedentary	3.07	(1.27–7.39) ^**^	3.32	(1. (22–9.03) ^**^
Medical history of NCDs^a^				
No	1	Reference	1	Re Reference
Yes	4.18	(2.30–7.61) ^***^	1.99	(1. (0.92–4.33)

^a^NCDs: non-communicable diseases;^b^Others: breakfast, lunch and supper; ^c^RTE: ready-to-eat; ^d^More frequently consumed (two to three times per day, once daily, two to four times per week, once per week and one to three times per month). ^e^Less frequently consumed (less than once per month) ^*^P,0.05;^**^P,0.01;^***^P < 0.001;OR: odds ratio, CI: confidence interval ^a)^ Adjusted for the variables listed in this table

## Discussion

To the best of our knowledge, this was the first study to identify the association between BMI and RTE food consumption among sedentary staff in Nay Pyi Taw Union Territory, Myanmar. It was found that frequent consumptions of RTE food were positively associated with overweight and obesity. In addition, the preference for eating RTE food during free time was nearly nine times more likely to contribute to overweight and obesity. In this study, respondents who were older, who did light-intensity physical exercise, who had sedentary leisure activities, and smokers were more likely to be overweight and obese than their counterparts.

In this study, the sedentary staff who consumed RTE food more than once per month were nearly five times more likely to be overweight and obese than those who did not. Sedentary staff mostly ate confections, carbonated beverages, instant noodles, and instant-mixes, which contained a large amount of calories, saturated fats, sugar, and salts. Food premises in public areas and canteens at workplaces facilitated workers to access RTE food easily. Respondents in this study were full-time workers working 8 h a day on weekdays. Sometimes, they had to work overtime on weekends. Thus, they did not spend much time in preparing home cooked meals. These factors caused them to consume RTE food more frequently than home-made meals. A study from the United States reported that frequent RTE food consumption led to increased energy intake, resulting in overweight and obesity [36]. This finding is in line with studies from Cameroon, Luxembourg, and Indonesia [37–39].

A similar finding was found in a Saudi study, which stated that frequent consumption of fast food was significantly associated with obesity [40]. Working people mainly consumed RTE food as they worked for long hours [41]. One study stated that full-time workers consumed fast food frequently due to convenience and tight working schedules, which led to obesity [42]. Therefore, the findings from this study suggest that awareness-raising campaigns on healthy dietary habits are urgently needed to be implemented in workplace settings. Regulatory authorities should strictly regulate food safety and food labeling. People should be aware of the nutritional contents of the food they consume.

This study also proved that the preference for RTE food consumption during free time was positively associated with overweight and obesity. In this study, 70% of respondents stored RTE food in their residences. This storage practice favors free time consumption of RTE food. Moreover, in this study, sedentary staff usually ate out at cafeteria for fun during their free time. They had easy access to fast food facilities in urban cities. Increased RTE food advertisements in mass media is one of the key factors for consuming RTE food [43]. This finding was supported by a study in the United States which reported that working women preferred RTE food due to limited time [44]. In addition, one study showed that people consumed RTE food because of its taste, ease of preparation good advertisement, and high income [45]. This study finding suggests that strict regulations should be developed against the over-branding of RTE food. Effective and sustainable health education on problems of unhealthy diet should target working people.

In this study, respondents over 32 years old were three times more likely to be overweight and obese than their counterparts. This may be partly due to the decreasing metabolic rate and lesser physical activity associated with aging. Other possible reasons for overweight and obesity were chronic NCDs such as ischemic heart diseases, hypertension, and type 2 diabetes mellitus in respondents. This study finding was similar to another study which reported that obesity prevalence was the highest in the adult population [46]. A study from Nepal showed that adults were more likely to be overweight [47]. Healthy lifestyle behavior should be adopted from younger ages to prevent metabolic diseases and obesity in adults.

This study showed that smokers were nearly six times more likely to be overweight and obese than non-smokers. The relationship between smoking and obesity was not clearly understood [48]. A study from Japan showed that smoking was associated with obesity, but the association depended on the number of cigarettes smoked, not on the duration of smoking. Short-term heavy smokers were more likely to be obese than long-term light smokers [49]. This study finding was contrary to another study which found that smoking was negatively associated with overweight and obesity [50]. Nicotine, being a cholinergic agonist, affects eating. Another possible reason is that smoking impaired the taste of the meal and reduced calorie absorption. A study from the United Kingdom mentioned that former smokers were more likely to be obese than current smokers and non-smokers [51]. In Myanmar, people smoked at smoke-free public areas as the Tobacco Control law is not strictly enforced in the country. Therefore, to reduce the risk of NCDs, the Tobacco Control Law in Myanmar should be strictly enforced.

The current study revealed that respondents who did physical exercises were less likely to overweight and obesity. Due to the modern fast-paced living, the nature of the work becomes more sedentary and people become physically inactive. High consumption of energy-dense food such as RTE food has more chance to be physically inactive lives. Sedentary work nature leads to reduced energy expenditure and increased risk of overweight and obesity. The low physical activity was associated with overweight and obesity [52]. This finding is consistent with studies done in Myanmar, Malaysia, and the United States [53–55]. Working environments should promote physical activity and integrating enough public spaces for physical exercise should be considered in urban development and planning.

This study indicated that respondents who had sedentary activities during their leisure time were more likely to be overweight and obese than their counterparts. Low energy expenditure due to sedentary lifestyles increases the risk of overweight and obesity. Sedentary staff would like to spend most of their time sitting, watching TV, using the internet, or playing games. Increased screen-time might result in overweight and obesity. One Indonesian study reported a significant positive association between sedentary activities and obesity [56]. A study conducted in India also proved that prolonged sedentary activities are important predictors of obesity [57]. Prolonged screen-time leads to passive snacking and drinking of beverages [58]. Highly sedentary behavior, such as watching television, is frequently associated with an increased risk of obesity [59].

There were some limitations in this study. Firstly, this study was conducted only in Nay Pyi Taw Union Territory, Myanmar. Thus, the findings may not be representative of the whole country. Secondly, the cross-sectional nature of the study could not find out the causal inferences. Thirdly, data collected on risk behaviors such as RTE food consumption, physical activities, sedentary lifestyle, smoking, and alcohol drinking could have subjective or recall bias as they were self-reported data. Fourthly, this study did not measure the central obesity of sedentary staff. Longitudinal studies with more detailed information should be performed in the future. This study is supposed to be the first study to identify the associations between BMI and RTE food consumption among sedentary staff in Myanmar.

## Conclusions

This study pointed out that frequent RTE food consumption and physically inactive lifestyles were the major predictors of overweight and obesity. Age, smoking, and sedentary leisure activities were positively associated with overweight and obesity. Effective and sustainable health promotion focusing on healthy dietary habits and active lifestyles should be implemented at workplaces. Every sedentary worker should be aware of the food-based dietary guidelines of the country. Policymakers should implement an effective and sustainable action plan on the compulsory labeling of nutritional information on the packaging of RTE food. Moreover, over-branding of RTE food should be strictly prohibited. This study highlights the urgent need for an awareness program on RTE food targeting the working community. Furthermore, the study findings suggest the working community to reduce sedentary activities, which in turn, will reduce the burden of obesity. It is strongly recommended that national level policies should be developed on healthy lifestyle promotion especially in workplace settings to become a healthy working community.

## Supplementary information

**Additional file 1.** English questionnaire.

## Data Availability

The datasets generated during and/or analyzed during the current study are available from the corresponding author on reasonable request.

## Abbreviations

AOR: Adjusted odds ratio
BMI: Body Mass Index
CI: Confidence Interval
NCDs: Non-communicable diseases
OR: Odds Ratio
RTE food: Ready-to-eat food
SPSS: Statistical Package of Social Science
WHO: World Health Organization
