# Supplementary material for: Association between body mass index and ready-to-eat food consumption among sedentary staff in Nay Pyi Taw union territory, Myanmar
Source: BMC Public Health. 2020 Feb 10;20:206. doi: 10.1186/s12889-020-8308-6 (PMC7011543; doi:10.1186/s12889-020-8308-6)
Supplement: Supplementary file 1 — Additional file 1. English questionnaire. [file 12889_2020_8308_MOESM1_ESM.pdf]

Body mass index and ready-to-eat food consumption among sedentary staff in Nay Pyi Taw Union Territory, Myanmar

English questionnaire

This questionnaire is only for research. The results will not be linked to individual respondent and will be kept confidential. Name of participant is not need to describe in detail. It is requested to answer all questions. Thank you for your participation.

Office use only

Code No. [ ]

Name of interviewer ______________________

Place of interview ______________________

Date of interview ______________________

Part A. Background characteristics of respondents

No	Questions	Coding Categories		Code
A1	How old were you at your last birthday?	Age (Completed years)<br>[ ][ ]		
A2	Sex	Male<br>Female	1<br>2	
A3	Designation	Senior officer<br>Mid-level officer<br>Officer<br><br>Others ------------------------<br>(Specify)	1<br>2<br>3<br><br>77	

A4	Marital status	Single<br>Married<br>Divorced<br>Separated<br>Widow<br><br>Others ------------------------<br>(Specify)	1<br>2<br>3<br>4<br>5<br><br>77	
A5	Do you have any children?<br>**(If absent, skip to question A7)**	Present<br>Absent<br>No response	1<br>2<br>99	
A6	If present, how many children do you have?	------------------------		
A7	Type of family	Nuclear<br>Extended<br><br>Others ------------------------<br>(Specify)	1<br>2<br><br>77	
A8	Monthly income of family (Kyats)	----------------------- (Kyats)		
A9	Do you usually travel on weekends and gazette holidays?	Yes<br>No<br>Don't know<br>No response	1<br>2<br>88<br>99	
A10	Distance between residence and market places (in miles).	----------------------[mile(s)]		
A11	Transportation facilities	On foot<br>Bike<br>Motorbike<br>Car<br>Public transport<br><br>Others ------------------------<br>(Specify)	1<br>2<br>3<br>4<br>5<br><br>77	

A12	Housemaid	Present<br>Absent<br>No response	1<br>2<br>99	
A13	Have you ever drunk liquor?<br>**(if not, skip to question A15)**	Yes<br>No<br>Don't know<br>No response	1<br>2<br>88<br>99	
A14	If drink liquor, how often did you drink?	Socially<br>Monthly<br>Weekly<br>Weekends<br>Daily<br><br>Others -----------------------<br>(Specify)	1<br>2<br>3<br>4<br>5<br><br>77	
A15	Have you ever smoke?<br>**(if not, skip to question A17)**	Yes<br>No<br>Don't know<br>No response	1<br>2<br>88<br>99	
A16	If smoked, how much cigarette/cigar/cheroot did you smoke per day?	Ten and above<br>Five to nine<br>One to four<br><br>Others -----------------------<br>(Specify)	1<br>2<br>3<br><br>77	
A17	Do you have a following diseases?<br>a. Hypertension<br><br><br>b. Ischemic heart diseases?<br><br><br><br>c. Diabetes mellitus	Yes<br>No<br>Don't know<br>No response<br><br>Yes<br>No<br>Don't know<br>No response<br><br>Yes<br>No<br>Don't know<br>No response	1<br>2<br>88<br>99<br><br>1<br>2<br>88<br>99<br><br>1<br>2<br>88<br>99	

A18	Do you do regular physical exercise?<br>**(if not, skip to A 21)**	Yes<br>No<br>Don't know<br>No response	1<br>2<br>88<br>99	
A19	Average physical exercise doing minutes per week.	--------------------- (Minutes)		
A20	What type of physical exercise do you do?	Walking<br>Swimming<br>Stretching<br>Cycling<br>Gym<br><br>Others ------------------------<br>(Specify)	1<br>2<br>3<br>4<br>5<br><br>77	
A21	What did you do in your leisure time?	------------------------		
A22	Cooking role for the family	Main role<br>Supportive role<br>Do not cook<br><br>Others ------------------------<br>(Specify)	1<br>2<br>3<br><br>77	

B. Ready-to-eat food consumption of respondents

No	Questions	Coding Categories		Code
B1	How frequent did you have ready-to-eat food?<br>**(A single response only)**	Never or less than once a month<br>One to three times per month<br>Once a week<br>Two to four times per week<br>Once per day<br>Two to three times per day<br>Four times and above per day<br><br>Others -----------------------<br>(Specify)	1<br><br>2<br>3<br>4<br>5<br>6<br>7<br><br>77	
B2	How frequent did you have instant noodle?<br>**(A single response only)**	Never or less than once a month<br>One to three times per month<br>Once a week<br>Two to four times per week<br>Once per day<br>Two to three times per day<br>Four times and above per day<br><br>Others -----------------------<br>(Specify)	1<br><br>2<br>3<br>4<br>5<br>6<br>7<br><br>77	
B3	How frequent did you have confections?<br>**(A single response only)**	Never or less than once a month<br>One to three times per month<br>Once a week<br>Two to four times per week<br>Once per day<br>Two to three times per day<br>Four times and above per day<br><br>Others -----------------------<br>(Specify)	1<br><br>2<br>3<br>4<br>5<br>6<br>7<br><br>77	

B4	How frequent did you have carbonated beverages?(non-alcoholic beverages)<br>**(A single response only)**	Never or less than once a month<br>One to three times per month<br>Once a week<br>Two to four times per week<br>Once per day<br>Two to three times per day<br>Four times and above per day<br><br>Others -----------------------<br>(Specify)	1<br><br>2<br>3<br>4<br>5<br>6<br>7<br><br>77	
B5	How frequent did you have instant mix?<br>**(A single response only)**	Never or less than once a month<br>One to three times per month<br>Once a week<br>Two to four times per week<br>Once per day<br>Two to three times per day<br>Four times and above per day<br><br>Others -----------------------<br>(Specify)	1<br><br>2<br>3<br>4<br>5<br>6<br>7<br><br>77	
B6	Do you have a habit of eating out?	No<br>Yes<br>Don't know<br>No response	1<br>2<br>88<br>99	
B7	Reason of eating out	For fun<br>For change<br>For family recreation<br>For saving time<br><br>Others -----------------------<br>(Specify)	1<br>2<br>3<br>4<br><br>77	
B8	Do you prefer for ready-to-eat food?	Yes<br>No<br>Don't know<br>No response	1<br>2<br>88<br>99	

B9	How long have you ate ready-to eat food?	More than ten years<br>Five to ten years<br>One to four years<br>Less than one year<br><br>Others ------------------------<br>(Specify)	1<br>2<br>3<br>4<br><br>77	
B10	When did you eat ready-to-eat food?	Breakfast<br>Lunch<br>Dinner<br>Supper<br><br>Others ------------------------<br>(Specify)	1<br>2<br>3<br>4<br><br>77	
B11	Do you usually eat ready-to eat on your free-time?	Yes<br>No<br>Don't know<br>No response	1<br>2<br>88<br>99	
B12	Do you use to buy and store ready-to-eat food in your home?	Yes<br>No<br>Don't know<br>No response	1<br>2<br>88<br>99	

C. Measurement of the body weight and height of the respondents

Height -------------------------- (cm)

Weight ------------------------- (kg)
